# Status of immunology education and establishment of American Association of Immunologists–recommended immunology content guidelines for undergraduate medical education in the United States

**DOI:** 10.1093/immhor/vlag041

**Published:** 2026-08-20

**Authors:** Justin Andrews, Timothy J Bauler, Andrea Bottaro, Jennifer T Grier, Pooja Jain, Vijaya Knight, Michelle Swanson-Mungerson, Scott B Thompson, Yuan Zhao, Nicholas A Pullen, Aimee Pugh-Bernard

**Affiliations:** University of Colorado Anschutz Medical Campus, Aurora, CO, United States; Western Michigan University Homer Stryker M.D. School of Medicine, Kalamazoo, MI, United States; Cooper Medical School of Rowan University, Camden, NJ, United States; University of South Carolina School of Medicine Greenville, Greenville, SC, United States; Drexel University College of Medicine, Philadelphia, PA, United States; University of Colorado Anschutz Medical Campus, Aurora, CO, United States; Chicago College of Osteopathic Medicine, Midwestern University, Downers Grove, IL, United States; University of Colorado Anschutz Medical Campus, Aurora, CO, United States; Sam Houston State University College of Osteopathic Medicine, Conroe, TX, United States; College of Natural and Health Sciences, University of Northern Colorado, Greeley, CO, United States; University of Colorado Anschutz Medical Campus, Aurora, CO, United States

**Keywords:** curriculum, immunology, medical education, medical school, teaching

## Abstract

Medical schools in the United States have varying curricular designs and limited instructional time, which challenge cohesive and comprehensive immunology coverage necessary for future physicians. To address this challenge, the American Association of Immunologists formed a Medical Education Immunology Curriculum Task Force, to generate recommendations for U.S. medical schools in the field of immunology. In June 2024, faculty from all U.S. medical schools were invited to participate in an initial research survey designed to assess the current field of immunology content taught to medical students. Approximately 30% of the invited participants responded, with 25% of respondents from DO programs and 75% from MD programs, which reflects the overall distribution of these schools nationally. This article shares the core content identified by the participating faculty, along with various demographic and opinion-based metrics. The Task Force developed specific learning objectives (LOs) and associated immune-relevant clinical conditions for each core area of content. These were then vetted by the broader medical immunology education community by presentation at an immunology-focused professional meeting and through a second feedback survey. The final recommended content includes foundational topics and associated LOs, as well as a list of key associated clinical conditions aligned with each foundational topic. These LOs have the advantage of being inserted throughout integrated curriculum and/or could be used in stand-alone immunology course blocks in the medical school curriculum. The inclusion of these LOs in a medical curriculum will sufficiently educate future physicians regarding the impact of the immune system on overall health and illness.

## Introduction

With the exponential growth of immunological knowledge, it is imperative that medical students receive early exposure to immunological topics and associated pathophysiology during their undergraduate medical education, the phase of medical training in which students complete the curriculum leading to an MD or DO degree, including both preclinical and clinical instruction. This knowledge is essential to build a solid foundation to integrate immune concepts and interactions across all areas of medicine. However, immunology as a stand-alone discipline and its importance as part of normal physiology may not be adequately covered due to the complexities of undergraduate medical education, the diversity of medical schools’ curricula, and the historical incorporation of immunology as part of microbiology. Furthermore, faculty also face difficult prioritization decisions about immunological content under curricular time constraints. Previous work has found that there is stark diversity in current immunology education across U.S. medical schools, ranging from course structure, curricular time allotted, and availability of upper division coursework.^1^

To further complicate the matter, medical education has been dramatically changing with shifts in curricular design. With the increasing focus in many medical schools on experiential clinical education, a shift toward active learning and reduction in preclinical curricular time, there are significant new time demands that limit immunology-centered instruction time. Furthermore, the shift toward an integrated curriculum, while beneficial for promoting interdisciplinary learning, can make consequential learning of complex foundational science more challenging. Previous traditional models of medical education emphasized 2 yr of preclerkship learning in classroom-based sessions designed to prepare students to take boards (USMLE step 1 and COMLEX level 1, for MD and DO programs, respectively),^2^ which are largely foundational science-focused exams. However, with the introduction of curricular models designed to train a more clinically focused physician, the amount of time dedicated to learning basic science information before clerkship has been compressed for an increasing number of schools, down to 18 mo or even 1 year.^3^

Throughout this rapid transformation in medical education and the field of immunology specifically, many immunology educators have been left to individually decide what topics to prioritize and how to introduce them within the unique framework of their preclinical medical school curriculum without much guidance or support. As a result of this lack of cohesion, medical students graduate with various levels of exposure to, and understanding of, immunology in the clinical sphere and its impact on patient care.

Furthermore, a 2022 review of the literature identified trends in pedagogy and other instructional influences that affect specialists in allergy and clinical immunology.^4^ For example, a notable shift in how foundational science content is offered (and how much) involves much greater emphasis on interprofessional education in the physician learner phases. In another study, it was found that active learning options such as preclass presentations, video and audio supplements, clicker questions, and concept maps could also further enhance student learning.^5^ This is in line with decades-old literature in active learning approaches for science education in fields such as physics, ecology, microbiology, and human physiology. This emphasizes a need for immunology to “catch up” with our peers, particularly in preclinical education (e.g. microbiology, etc.). In other words, there is a need for a recommendation of fundamental content in the context of various pressures on instructional time, student preparation, and learner engagement.

Unlike undergraduate baccalaureate education, medical school has a narrower objective: to prepare future physicians that have mastered the fundamental topics of immunology to succeed in providing basic patient care. Additional training after medical school, such as allergy/clinical immunology or rheumatology fellowships, may be pursued to enhance immunological knowledge. However, the core immunologic topics still arise within and impact every field of medicine, from primary care to specialty disciplines, since the immune system is mobile and interacts with every system in the body.

Further compounding these issues is that most medical students’ first extensive exposure to immunology is in medical school; immunology is not a prerequisite for medical school admission.^1^ Formal immunology coursework is rarely required for biology degrees, except as an upper-division elective. It is often not offered when there is a gap in immunology teaching specialty within the faculty of an undergraduate baccalaureate biology department. Additionally, unlike other areas in undergraduate baccalaureate degrees, such as biochemistry or neuroscience, immunology does not regularly have its own track. While many students in baccalaureate microbiology or general physiology courses receive some exposure to immunology, the content amount, depth, and breadth are often determined by individual instructors’ specialty.^6^ Even when formal baccalaureate immunology coursework is provided, there is a wide range in the diversity of topics taught and inconsistencies between institutions persist.^7^ This lack of cohesion and structure in undergraduate immunology education directly affects students pursuing medical education. This highlights the ongoing need for curricular standardization and early introduction of immunology in undergraduate baccalaureate programs to motivate students’ interest and provide a foundational understanding of immunology concepts to students interested in health care careers.^6–8^

The needs identified from the examination of undergraduate baccalaureate immunology education have led in recent years to the creation of American Association of Immunologists (AAI)–endorsed content recommendations.^8^ In contrast, no specific guidelines currently exist for fundamental immunology content in undergraduate medical education. In addition to the necessity of topic guidelines, the impact of core learning objectives (LOs) in medical education has been demonstrated.^9^ For example, in one study, researchers found that creating LOs and emphasizing their usage in medical students’ physiology coursework allowed for increased levels of active learning and student engagement.^10^ Furthermore, clear and meaningful LOs appeared to significantly contribute to students’ learning.^11^^,^^12^ Therefore, topic recommendations with corresponding LOs would be beneficial not only for faculty, but ultimately for student understanding. To address this gap, the AAI Council and AAI education committee created an ad hoc Medical Immunology Curriculum Task Force, to investigate how immunology is taught to medical students throughout the preclinical and clinical years and to issue recommendations on foundational topics to be covered during undergraduate medical education. Furthermore, developing LOs is considered a best practice, as it promotes consistency within diverse medical school curricula, supports flexibility and integration across multiple curricular models, and ensures transparency and tracking in relation to content-based assessment items. Finally, this information can be used at individual institutions as part of their ongoing undergraduate medical education immunology curriculum assessments and comparisons to peers.^13^

In the present paper, we establish AAI-recommended immunology content for undergraduate medical education. Our results include analyses of demographic information from a medical educator community survey that provides valuable insights into the state of teaching medical immunology in the United States as of 2025. Although approximately 30% of invited participants responded, the sample achieved strong representativeness: 29% of respondents were from DO programs and 71% from MD programs, closely mirroring the national distribution of these schools. This alignment indicates that the dataset reached saturation with respect to program type. The final products of this study are the fundamental immunology content recommendations, LOs, and aligned immune clinical conditions for use as potential case examples. Together, these resources provide a practical framework to guide curriculum development, standardize foundational immunology education, and support the preparation of future physicians.

Like the fast-paced field of immunology, these medical immunology curriculum recommendations are dynamic and will be updated periodically. Review and updates will be conducted at regular intervals by the AAI through members of the Science and Education Core Committee and specific stakeholders in medical immunology teaching. Any updates to the curriculum recommendations will be housed with the web presence of the AAI Science and Education Core Committee or as designated by the AAI.

## Methods

**Figure 1:**
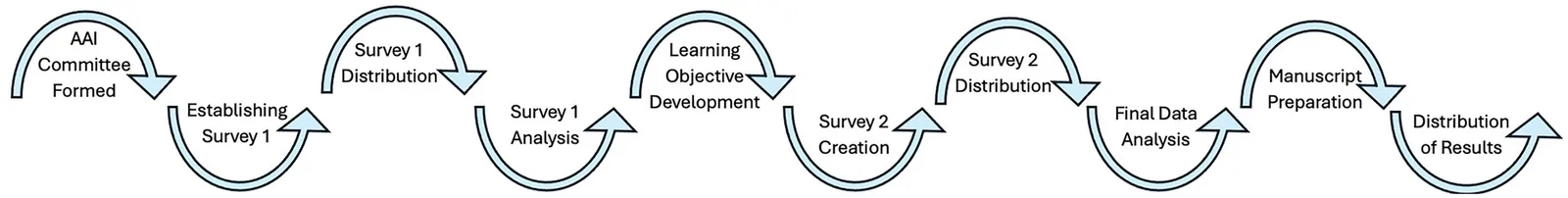
Process of undergraduate medical education immunology curriculum recommendation development. AAI, American Association of Immunologists.

The process for developing AAI-endorsed content recommendations is outlined in Figure 1 and summarizes the multistep process in the development of these recommendations that took place from December 2023 to December 2025. To assess the presentation of medical schools’ immunology curriculum, a primary survey (survey 1, Fig. S1) was created and sent to identified contacts of all MD and DO accredited and preaccredited U.S. medical schools as of June 2024. The identification of possible contacts was conducted for each MD and DO school through the following process using internet searches: immunology teaching faculty or relevant course director, then when such a person could not be identified a department chair, and finally dean’s office staff involved in administering preclinical education. For each contact, their name, institution, email address, and specific notes about their qualifications for receiving the survey were recorded. For institutions where multiple contacts were easily identified, their information was also recorded as well in case the primary contact could not be reached. This hierarchical and standardized approach was used to minimize misclassification and to maximize the likelihood that the survey was received by individuals with direct knowledge of their institution’s immunology curriculum. This information was kept in a spreadsheet and was only accessible by members of the Task Force. Contacts were reviewed independently by each member of the Task Force. Each identified primary contact was emailed the primary survey and instructed to complete the survey before data capture was terminated by the lead investigators. Participants were asked to complete demographic questions and indicate whether each of a set of basic immunology topics, identified by the Task Force through a review of commonly taught content across undergraduate medical curricula, national examination blueprints, and high-yield board-preparation resources, is covered in their curriculum. The period of data collection was from June 2024 through July 2024. The survey was created and distributed by Qualtrics, and primary data capture was completed through this platform. Analysis of these initial results from survey 1 were presented at the 2025 annual AAI conference in the Immunology Teaching Interest Group session, as well as a separate poster in the scientific session.^14^

Upon completion of the data collection, the Task Force met to discuss the results of the survey and begin drafting LOs for the 15 foundation topics highlighted in survey 1. These topics include cells, organs and microenvironments of the immune system; innate immunity and inflammation; the complement system; adaptive immunity: B cells—development, activation, and effector function; adaptive immunity: antibody structure and function; adaptive immunity: MHC—antigen processing and presentation; adaptive immunity: T cells—development, activation, and effector function; host-pathogen interactions; vaccines: formulations and immune response; hypersensitivities; transplantation immunology; tumor microenvironment and immune response; tolerance and autoimmunity; immunodeficiency; and serology and immunologic assays.

The Task Force then produced foundational topic-aligned LOs and highlighted immunological conditions for clinical context and/or case-based teaching. Feedback from the community of immunology educators was collected first by a standalone session at the 2025 annual AAI conference in which draft LOs were provided to attendees for real-time feedback and discussion. An additional window for feedback was then provided until June 2025 for attendees to respond at length via editing of a shared document and direct email. Finally, feedback from the meeting attendees was incorporated and the LOs were then redistributed to survey 1 contacts for further feedback and editing via survey 2 (Fig. S2). This second community survey asked respondents to provide editorial feedback on the content recommendations provided in Tables 1 and 2. After collecting feedback, the content tables were further edited for clarity by the Task Force and presented herein. The relative distribution of the location of respondents for both survey 1 and survey 2 are shown in Figure S3. All data were stored in Microsoft Excel workbooks. Descriptive statistics were calculated for each Likert scale item to summarize response patterns. Institutional Review Board Exemption for this research was granted by each author’s University Institutional Review Board Committee for this study.

**Table 1:** Foundational topics and learning objectives for immunology medical education.

Foundational topic	Learning objectives
Cells, organs, and microenvironments of the immune system	Recognize overarching concepts that provide the structural framework for the function of the immune system Differentiate innate versus adaptive components of the immune system Differentiate between myeloid and lymphoid cell types Define terminology such as antigen, epitope, the antigen receptors, clonal selection and expansion, naive and effector, antibody, antigen presentation, and memory formation Align key term/functions with major cell types Describe the functions of the primary/central tissues of the immune system Describe the functions of the secondary/peripheral tissues of the immune system, including mucosal-associated tissues Correlate the anatomy of spleen and lymph nodes with their functions in an adaptive response Analyze the role of the lymphatic system in the transport of antigen and immune cells within the body Define different types of cell death and inflammatory factors Define immunosenescence and the associated changes in immune system function across the human lifespan
Innate immunity and inflammation	Identify and describe the functions of physical and chemical barriers to infection Explain the role of pattern recognition receptors and describe how innate immune responses are triggered in the context of pathogens and damage Compare/Contrast the events of an inflammatory process in response to injury and infection Summarize the chemical components, soluble proteins, and cell types involved in innate immune responses and their roles in the inflammatory process or infection response Describe the process of leukocyte extravasation Describe the process and enzymes involved in phagocytic killing of microbes Explain the functions of natural killer cells and significance of major histocompatibility complex in these actions Evaluate the role of inflammatory cytokines and chemokines in inflammatory responses Describe the significance of the innate immune response in the activation of the adaptive immune response Compare and contrast acute and chronic inflammation Define autoinflammation and distinguish it from autoimmunity by describing the innate immune mechanisms and clinical features involved, including the structure and function of inflammasomes and their role in autoinflammatory disease Outline mechanisms of resolution of the inflammatory response
The complement system	Describe the 3 pathways of complement activation, including convergence of the pathways Describe the principal biological functions of complement List the mechanisms of action and/or target of the regulatory proteins (including inhibitors) and receptors of complement Summarize how key fragments (e.g. C3b, C3a, C4a, C5a, C5b) drive opsonization, chemotaxis, inflammation, lysis, and immune-complex and altered cell clearance Describe evasion tactics that inhibit or hijack complement (e.g. microbes, cancer cells)
Adaptive immunity: B cells—development, activation, and effector function	Describe the role of the bone marrow in the maturation and selection of B lymphocytes Explain the process of V(D)J recombination (aka “somatic recombination”) in relation to the rearrangement of the B cell receptor and development of a B cell Explain the molecular mechanisms and functional significance of the modifications that occur in the germinal center Describe the process of B cell activation Compare and contrast T-dependent and T-independent B cell responses Explain the concept of “linked recognition” between a B cell and T cell for activation Compare and contrast naïve and memory B cell responses Compare the roles of cell-mediated and humoral immunity
Adaptive immunity: antibody structure and function	Diagram and describe the structural components of a typical membrane bound and secreted immunoglobulin (antibody) Describe the effector functions of antibodies Describe the role of the antibody Fc region in terms of their effector functions Describe the role of the antibody Fab region in terms of antigen binding Compare and contrast immunoglobulin isotypes (classes) in the context of structure, function, and location Compare and contrast the clinical usefulness of monoclonal versus polyclonal antibodies Explain the biochemical interactions (e.g. noncovalent forces) between antibody and antigen and the effect on binding Diagram and describe antibody complementarity determining regions (CDRs, aka “hypervariable region”) and their roles in antigen binding Explain the concept of clonal selection in response to antigen Describe the mechanism and functional consequences of somatic hypermutation and isotype switching
Adaptive immunity: MHC—antigen processing and presentation	Define major histocompatibility complex and HLA Compare the genes, structure, cellular expression, peptide display and function of major histocompatibility complex classes I and II Describe the biological significance of peptide display via major histocompatibility complex class I and II molecules Compare the mechanisms of antigen processing, presentation and recognition for endogenous and exogenous antigens Evaluate the association of HLA with certain diseases
Adaptive immunity T cells—development, activation, and effector function	Explain the process of V(D)J recombination (aka “somatic recombination”) in relation to the rearrangement of the T cell receptor and development of a T cell Describe the process of T cell development Explain the basic structure of T cell receptors and major histocompatibility complex interaction Compare and contrast the generation and activation of naïve T cells and memory T cells Explain the functions of effector T cells, including cytotoxic T cells and T helper cell subsets (T helper 1, T helper 2, T helper 17, T follicular helper, T regulatory)
Host-pathogen interactions	Explain circumstances in which microbes cause acute versus chronic infection and develop inflammation Summarize the cellular, molecular, and chemical mechanisms by which pathogens are detected by the immune system Distinguish different mechanisms microbes employ to evade immune defenses Identify immune responses that are employed to attack intracellular versus extracellular pathogens Summarize the overarching concepts of immune defense including detection and recognition, elimination, tolerance, and/or immune memory Describe the formation of a granuloma
Vaccines: formulations and immune response	Compare passive immunization to active immunization Evaluate vaccines in relation to formulation and immune response Explain how vaccines generate immune memory and why it is essential for long-term protection Review clonal selection in relation to immunization Describe the function of vaccine additives Define herd immunity (aka “community immunity”) Discuss the use of vaccines in populations with immunodeficiency or an immunocompromised state (aka “altered immunocompetence”) Explain the scientific rationale behind the production of the annual influenza vaccine Define vaccine hesitancy and develop an approach to vaccine-hesitant patients Identify vaccine associated adverse events
Hypersensitivities	Define immunological hypersensitivity and compare/contrast with non-immunological hypersensitivities Describe the immunological mechanism(s) and clinicopathological manifestations of common forms of type I hypersensitivity Describe the immunological mechanism(s) and clinicopathological manifestations of common forms of type II hypersensitivity Describe the immunological mechanism(s) and clinicopathological manifestations of common forms of type III hypersensitivity Describe the immunological mechanism(s) and clinicopathological manifestations of common forms of type IV hypersensitivity
Transplantation immunology	Define types of transplants based on genetic similarity: autologous, syngeneic, allogeneic, and xenogeneic Explain the role of major histocompatibility complex/HLA and other alloantigens in the process of graft acceptance or rejection Summarize the processes of T cell recognition of foreign antigen within grafts Describe the mechanisms that contribute to distinct types of graft rejection Explain immunomodulatory treatment strategies for the prevention of graft rejection Describe the difference between solid organ transplantation and hematopoietic stem cell (bone marrow) transplantation with respect to HLA matching and graft-versus-host disease Explain the immunological mechanism of blood group incompatibility and transfusion reactions
Tumor microenvironment and immune response	Describe immunosurveillance and the role of immune cells in antitumor immunity Identify different types of tumor antigens Explain immunoediting and the ways in which tumors evade immune recognition Describe the role of the tumor microenvironment in antitumor immunity and immune escape Assess immunotherapeutic strategies to harness the immune system for cancer treatment Identify new immunotherapies designed to target cancer and autoimmune conditions
Tolerance and autoimmunity	Define immunological tolerance, and compare/contrast the terms “autoreactivity,” “autoimmunity,” and autoimmune disease Compare and contrast B and T cell central and peripheral tolerance Explain positive and negative T cell selection in the thymus, and their significance for the development of the T cell repertoire and the establishment of central T cell tolerance Discuss the development of T-regulatory cells and explain their role in peripheral tolerance Describe some of the factors that contribute to the development of autoimmunity and autoimmune disease Examine the range of immunopathological mechanisms involved in autoimmune disease and link them to different types of hypersensitivity Explain the significance of elucidating the immunopathological mechanisms of autoimmune diseases for their diagnosis, prognosis, and targeted treatment Describe the basic immunologic mechanism(s) and immunotherapeutic treatments associated with systemic and organ-specific immune-mediated disease Identify the role of immunomodulators, disease-modifying antirheumatic drugs, kinase inhibitors, and biologics in treatment of autoimmune conditions
Immunodeficiency	Discuss the diversity, presentation, and epidemiology of inborn errors of immunity/primary immunodeficiencies Describe the classifications of inborn errors of immunity and explain the key clinical, genetic, and pathophysiological features of immunological abnormalities arising from defects in T cells, B cells, phagocytes, and complement Develop and justify a clinical approach to the evaluation and diagnosis of a patient with suspected inborn errors of immunity based on: patient/family history, physical exam, and laboratory studies Outline key aspects of the management and treatment of immunodeficient patients Discuss examples of acquired (secondary) immunodeficiency diseases, identifying common causal mechanisms (e.g. infections, pharmacological/iatrogenic causes, toxicity, malignancy, trauma, aging, malnutrition)
Serology and immunologic assays	Define titer, antiserum, antiglobulin, and agglutinin Describe the impact of specificity and sensitivity on the interpretation of an assay For the antibody-mediated assays covered, describe the principal behind the assay, whether the assay is quantitative or qualitative, is highly specific or sensitive, source of material for the assay, advantage/disadvantage of the assay, and the clinical applications For the cell-mediated assays covered, describe the principal behind the assay, whether the assay is quantitative or qualitative, is highly specific or sensitive, source of material for the assay, advantage/disadvantage of the assay and the clinical applications For complement-mediated assays, explain what the CH50 and AP50 assays measure and how they are interpreted

**Table 2:** Foundational topics and associated conditions for immunology medical education.

Foundational topic	Associated conditions
Cells, organs, and microenvironments of the immune system	Lymphatic filariasis Distribution of cancer spread via lymphatics Edema after significant lymph node dissection Increased risk of encapsulated bacterial infection after splenectomy
Innate immunity and inflammation	Leukocyte adhesion deficiency Chronic granulomatous disease Gout Sarcoidosis Inflammatory bowel disease (ulcerative colitis, Crohn’s disease) Chediak-Higashi syndrome Periodic fever syndromes (e.g. familial Mediterranean fever) Systemic inflammatory response syndrome and cytokine storm reactions (e.g. septic and toxic shock, hemophagocytic lymphohistiocytosis)
The complement system	Early complement deficiencies (C1–C4) C1q, C2, C1 esterase deficiencies Deficiencies in C3 with recurrent pyogenic infections Hereditary angioedema (C1-INH deficiency) Late complement deficiencies (C5–C9) C5–C9 deficiency with increased risk for *Neisseria* species Paroxysmal nocturnal hemoglobinuria (defective CD55, CD59)
Adaptive immunity: B cells—development, activation, and effector function	X-linked agammaglobulinemia Common variable immunodeficiency Hyper-IgM syndrome
Adaptive immunity: antibody structure and function	Systemic lupus erythematous Type 1 diabetes Graves’ disease Hashimoto’s thyroiditis Celiac disease Rheumatoid arthritis Psoriasis Multiple sclerosis Myasthenia gravis Lambert-Eaton Vitiligo Autoimmune hemolytic anemia (warm and cold) Sjogren syndrome Scleroderma Limited scleroderma (CREST) syndrome Goodpasture syndrome Granulomatous with polyangiitis Eosinophilic granulomatosis with polyangiitis Microscopic polyangiitis Pemphigus vulgaris Bullous pemphigoid Hyper-IgE syndrome (Job syndrome) Hyper-IgM syndrome
Adaptive immunity: MHC—antigen processing and presentation	Bare lymphocyte syndromes (I and II) HLA subtypes with associated disease: B27: psoriatic arthritis, ankylosing spondylitis, IBD arthritis, Reactive arthritis DQ2/DQ8: celiac disease DR3: Type 1 diabetes, systemic lupus erythematosus, Graves, Hashimoto thyroiditis, Addison’s disease DR4: Rheumatoid arthritis, type 1 diabetes, Addison’s disease
Adaptive immunity T cells—development, activation, and effector function	Severe combined immunodeficiencies Newborn screen Omenn syndrome Autoimmune lymphoproliferative syndrome Autoimmune polyendocrine syndrome (APS-1, autoimmune polyendocrinopathy-candidiasis-ectodermal dystrophy) Immune dysregulation polyendocrinopathy enteropathy X-linked (IPEX) syndrome DiGeorge syndrome Hyper-IgE syndrome (e.g. STAT3 deficiency)
Host-pathogen interactions	Common viral infections Influenza Herpes viruses Hepatitis viruses HIV SARS-CoV2 (COVID-19) Measles Common bacterial infections and immunology interactions *Staphylococcus aureus* *Streptococcus pneumoniae* Tuberculosis *Neisseria* *Pseudomonas aeruginosa* *Escherichia coli* Common Parasitic Infections *Giardia lamblia* *Cryptosporidium* *Toxoplasma gondii* *Plasmodium* (malaria) Nematodes Cestodes Common fungal infections and host interaction Candida albicans Aspergillus fumigatus *Pneumocystis jirovecii* pneumonia Histoplasmosis Blastomycosis Coccidiomycosis
Vaccines: formulations and immune response	Anaphylaxis
Hypersensitivities	Type I hypersensitivities Hay Fever Anaphylaxis Allergic asthma Food and drug allergies Type II hypersensitivities Cellular destruction Autoimmune hemolytic anemias Immune thrombocytopenia Transfusion reactions Hemolytic disease of the newborn Inflammation Goodpasture syndrome Rheumatic fever Hyperacute transfusion reaction Cellular Dysfunction Myasthenia gravis Graves’ disease Pemphigus vulgaris Type III hypersensitivities Serum sickness Arthus reaction Systemic lupus erythematous vasculitis, arthritis, nephritis Polyarteritis nodosa IgA vasculitis Poststreptococcal glomerulonephritis Type IV hypersensitivities Contact dermatitis (e.g. poison ivy and nickel) Drug reaction with eosinophilia and systemic symptoms Stevens-Johnson syndrome Tuberculin skin test Graft-versus-host disease
Transplantation immunology	Hyperacute transplant rejection Acute transplant rejection Chronic transplant rejection Graft-versus-host disease Transfusion-related acute lung injury Delayed hemolytic transfusion reaction
Tumor microenvironment and immune response	Melanoma Leukemia Lymphoma
Tolerance and autoimmunity	Systemic lupus erythematous Type 1 diabetes Grave’s disease Hashimoto’s thyroiditis Celiac disease Rheumatoid arthritis Psoriasis Multiple sclerosis Myasthenia gravis Lambert-Eaton Vitiligo IgA deficiency Poststreptococcal glomerulonephritis Rheumatic fever Autoimmune hemolytic anemia (warm and cold) Sjogren syndrome Scleroderma Limited scleroderma (CREST) syndrome Goodpasture syndrome Granulomatous with polyangiitis Eosinophilic granulomatosis with polyangiitis Microscopic polyangiitis Bullous pemphigoid Pemphigus vulgaris Guillain-Barré syndrome Dermatomyositis Polymyositis
Immunodeficiency	B cell disorders X-linked agammaglobulinemia Selective IgA deficiency Common variable immunodeficiency T cell disorders Thymic aplasia (DiGeorge syndrome) IL-12 IFNy axis deficiencies (Mendelian susceptibility to mycobacterial disease) Hyper IgE syndrome (Job syndrome) Chronic mucocutaneous candidiasis B and T cell disorders Severe combined immunodeficiencies Ataxia-telangiectasia Hyper-IgM syndrome Wiskott-Aldrich syndrome Phagocyte dysfunction Leukocyte adhesion deficiency Chediak-Higashi syndrome Chronic granulomatous disease Early complement deficiencies (C1–C4) C1q, C2, C1 esterase deficiencies Deficiencies in C3 with recurrent pyogenic infections Hereditary angioedema (C1-INH deficiency) Late complement deficiencies (C5–C9) C5-C9 deficiency with increased risk for *Neisseria* species Paroxysmal nocturnal hemoglobinuria (CD55, CD59 deficiency)

Note. To ensure thorough and comprehensive coverage, some conditions may appear in multiple categories within this table, reflecting their relevance to more than 1 foundational immunology topic.

## Results

### Survey response analysis

As mentioned previously, our initial survey was to determine both the presentation and breadth of immunology topics throughout the U.S. medical schools (see supplementary files for survey 1). Out of 210 surveys distributed, 86 responses were recorded, 65 of which were considered complete and 16 of which were considered incomplete due to not all the survey questions being answered, but answers provided were included in the final analysis. The remaining 5 responses were duplicated responses from the same surveyor or multiple responses from the same institution and were excluded from the demographic analysis. The overall response rate was therefore approximately 30%, consisting of about 29% DO and 71% of MD school respondents, which is roughly comparable to the national numbers of DO and MD schools as of January 2025 (46 and 160, respectively).^15^^,^^16^

### Medical school immunology pedagogy analysis

**Figure 2:**
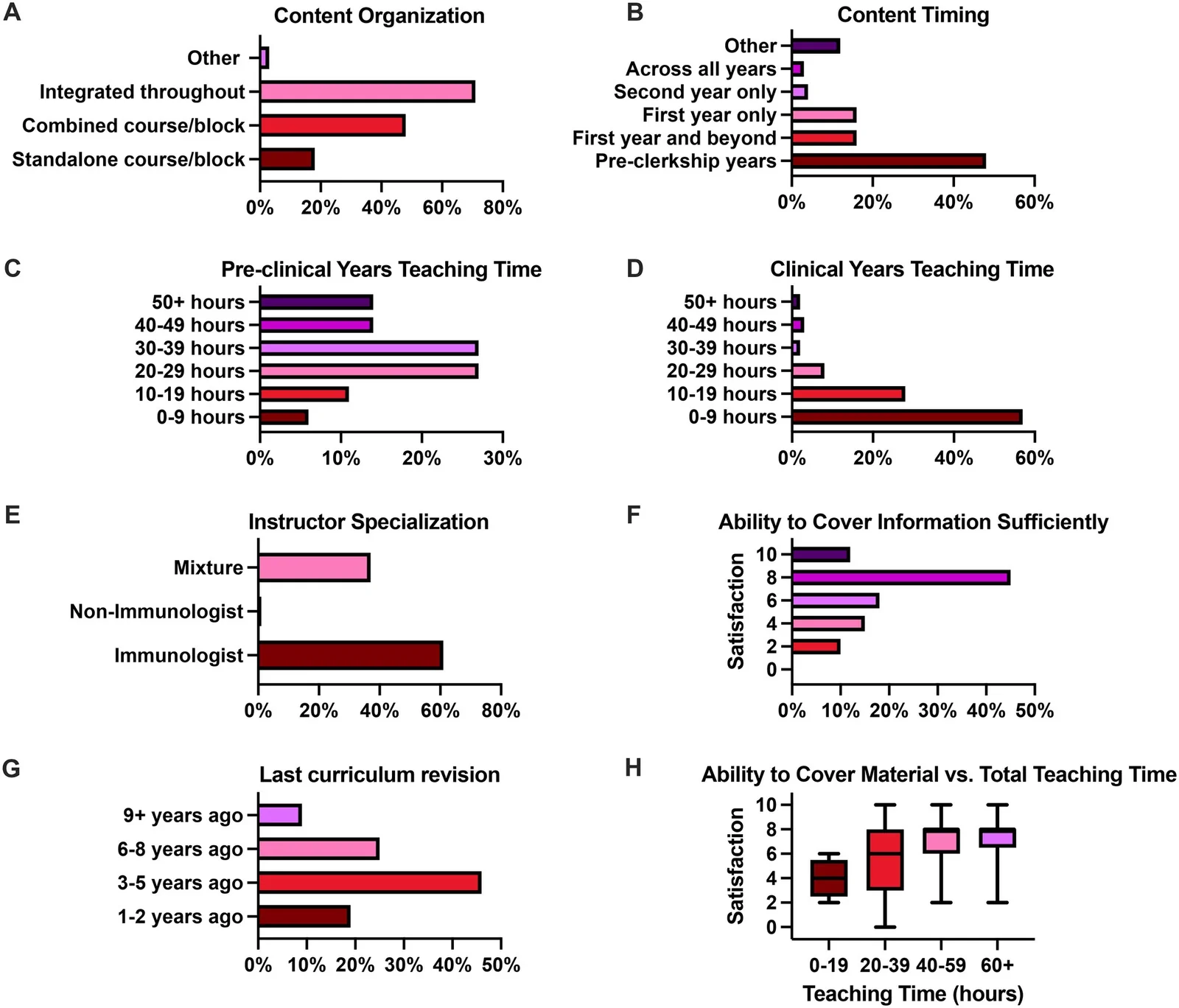
Overview of Organization and Sequencing of Immunology Content Responses as percentage of total for the following survey questions: A) How is the immunology content delivered in your curriculum? Select all that apply. B) When is the immunology content delivered? C) How much instructional time is devoted to immunology content in the pre-clinical or D) clinical years? E) Who covers foundational immunology teaching at your institution? F) Using a scale from 0 (not at all sufficient) to 10 (more than sufficient), do you feel that you can cover essential immunology content in the time that you are provided? G) When was the last time your institution substantially renovated the medical school curriculum? H) Box plot of satisfaction with time to successfully cover content (0, not at all sufficient, to 10, more than sufficient) versus estimated combined pre-clinical and clinical immunology teaching hours. Bars represent the maximum and minimum values; box ends are the first and third quartiles with middle line indicating the median.

As part of our survey, the delivery and content of immunology was assessed for the respondent medical schools. With the ability to indicate multiple delivery format responses, 71% of respondents indicated that the content was integrated throughout the curriculum, 48% in a combined course/block, 18% as a stand-alone course/block, with 3% using other formats (Fig. 2A). When asked about the time devoted to immunology content in the preclinical years, most respondents fell within ranges of either 20 to 29 h (27%) or 30 to 39 h (27%) of instructional time (Fig. 2B). Of note, a smaller but not insignificant number of programs (17%) dedicate <20 h of preclinical time to immunology content, while 14% of schools report having >50 h of immunology instruction time (Fig. 2C). In contrast to the preclinical curriculum, much less time was dedicated to immunology content during the clinical years. Most respondents (58%) stated that immunology was only given 0–9 h of instructional time in the clinical years, and another 28% reported anywhere between 10–19 h of dedicated curriculum time (Fig. 2D). Given the wide variation in curricular time allotted to immunology instruction, we asked respondents to assess, on a scale of 0 to 10, where 0 is not at all sufficient and 10 is more than sufficient, whether they felt that the time allotted for immunology in their programs was appropriate to cover the content in their courses, most respondents rated the amount of time given as 6 (18%), 8 (45%), or 10 (12%), indicating overall satisfaction with available time. However, 25% of respondents found the time allocated insufficient as either a 2 of 10 (10%) or a 4 of 10 (15%) on the Likert scale. These findings suggest that from the perspective of faculty respondents, curricular time devoted to immunology may be inadequate in one-quarter of schools surveyed (Fig. 2F). To further analyze the amount of time necessary for teaching a modern immunology curriculum we summed respondents’ estimates of both preclinical and clinical immunology hours and divided the response into 20-h ranges. We then plotted the corresponding respondents’ satisfaction score (from Fig.2F) as a box plot for each of these estimated total immunology teaching time ranges (Fig.2H). This visual comparison depicts low satisfaction below 20 h, higher satisfaction above 40 to 59 h, with only a minimal further increase at 60+ h (Fig. 2H). The majority of the respondents reported the timing of the most recent curriculum revision occurred within the last 3 to 5 yr (Fig. 2G). This information could be used by immunology educators to better understand the typical timing and frequency of medical school curriculum updates across the country. Given the rapid pace of scientific discovery, particularly in immunology, frequent updates would ensure that future physicians receive foundational training that reflects the evolving landscape of immune-mediated diseases and novel immunotherapeutics.

Regarding the specific background of medical school immunology instructors, most programs (61%) indicated that they had a trained immunologist teaching medical students throughout their education, and almost all the other respondents (37%) reported having a mixture of trained immunologist(s) and nonimmunologist(s) as instructors. Only 1 respondent reported not having a trained immunologist as primary instructor for their immunology curriculum (Fig. 2E). The respondents were then asked to provide an estimate of whether their students had had substantial exposure to immunology prior to beginning medical school. A large majority of respondents (84%) estimated that <50% of their students had substantial prior exposure to immunology, reflecting their perceptions rather than data from a formal assessment.

### Immunology curriculum content analysis

**Figure 3:**
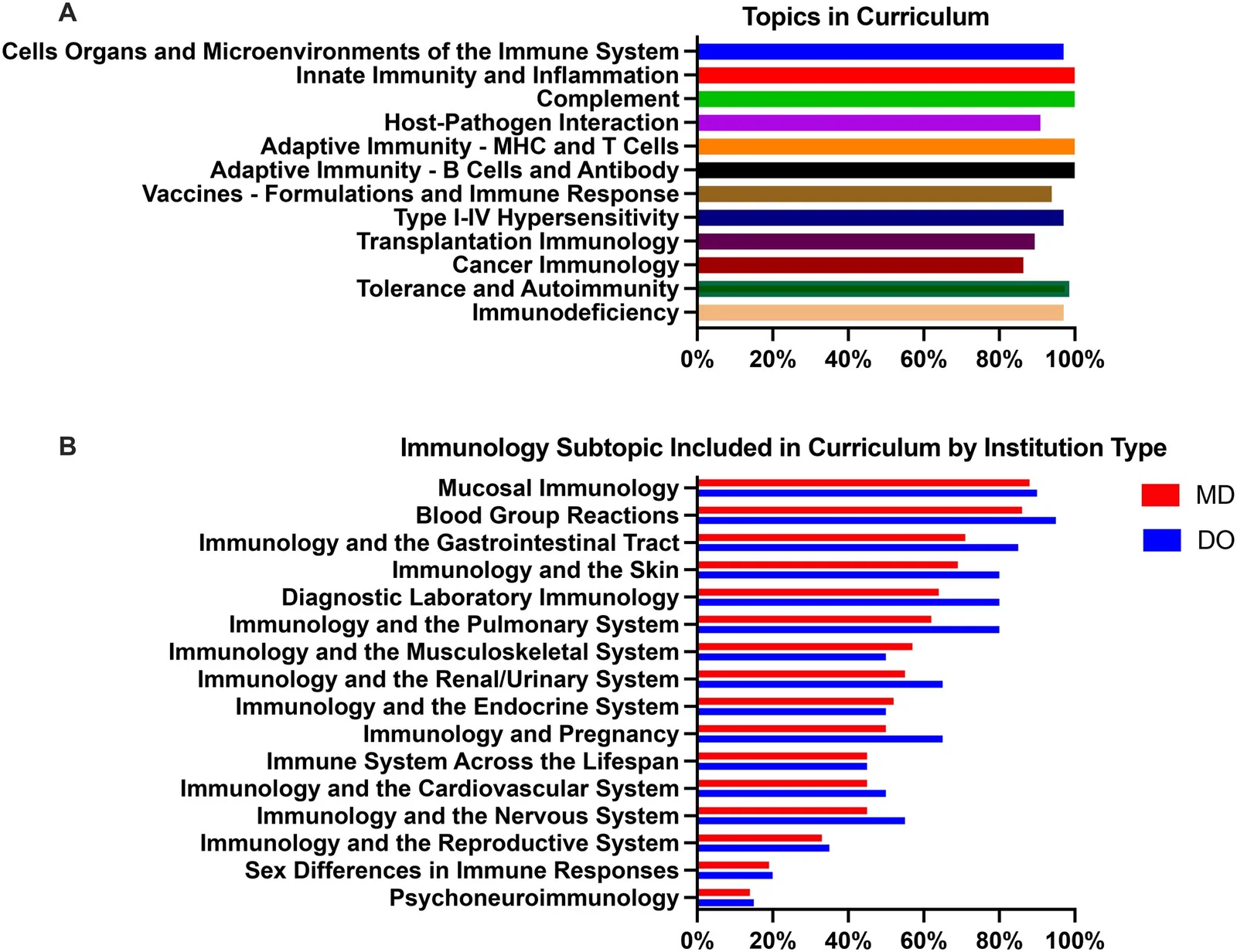
Overview of Immunology Topic Coverage A) Percentage of respondents indicating that a foundational immunology topic is included in the curriculum. B) Percentage of respondents from institutions granting either M.D. (red) or D.O. (blue) degrees indicating that an immunology subtopic is included in the curriculum.

Regarding specific topics covered in immunology preclinical medical instruction, there was a very large overlap of major subject areas between schools (Fig. 3). Greater than 90% of respondents reported covering a range of topics from basic (e.g. cells, organs, and microenvironments of the immune system, and B and T cell adaptive immunity) to more clinically relevant concepts (e.g. vaccines and hypersensitivities, tolerance and autoimmunity, and immunodeficiency). More advanced/specialized topics such as transplant immunology and cancer immunology were also largely covered, by between 80% and 90% of medical schools surveyed (Fig. 3A). Finally, respondents were tasked with selecting from a list of additional topics/content areas as well as subtopics potentially covered in their respective institutions’ curricula. While some subtopics were represented to a high degree among respondents (e.g. mucosal immunology, blood group reactions), other subtopics were minimally represented (e.g. sex differences in the immune response and psychoneuroimmunology). Both MD and DO schools had similar response coverage of these additional subtopics (Fig. 3B).

### Development of curriculum content recommendations

Through analysis of the topics and subtopics described in previous sections of the survey, specific LOs were produced and aligned into a total of 15 foundational topics (see Table 1). Where applicable, specific immunology-associated clinical conditions were also assigned to each of these foundational topics (see Table 2). These LOs and conditions were then returned to initial respondents in the form of a secondary survey for feedback and modification (see survey 2, Fig. S2).

For the LO list, respondents were asked to evaluate, on a scale from 1 to 5, how completely the objectives were covered at their institution, as well as if they believed that the objectives sufficiently covered essential immunological content for undergraduate medical education. Responses scored the proposed objectives an average of 4.2 in terms of current coverage within institutions, and of 4.8 in terms of comprehensiveness (see Fig. S4). Respondents were also provided an opportunity for free comments and recommendations for further edits to the language of the content objectives, which were considered for the final version presented here. Last, for this secondary survey, respondents indicated that society-endorsed content recommendations for immunology teaching in medical school would be of help toward the development and continuing revision of immunology curriculum in medical schools, scoring an average 4.6 on a scale from 1 (*not helpful*) to 5 (*very helpful*).

## Discussion

In an era of misinformation regarding vaccines and other immunology-related topics, physicians remain one of our most trusted resources when it comes to patients’ health.^17–19^ Considering this, ensuring that current and future physicians have a strong foundation in immunology is becoming increasingly important. The data presented herein surveyed medical educators nationally to provide a contemporary snapshot of how immunology is presented in their curriculum and gathered information to highlight relevant topics for inclusion in medical school curricula to ensure physicians are adequately trained in immunology.

One of the most interesting findings was the significant variation in immunology curriculum time and distribution within the curriculum among U.S. medical schools (see Fig. 2). While this is not unexpected, based on the diversity of curricular designs, distribution of didactic approaches, and overall time allocated for preclinical education in different medical schools, it highlights the need for guidance regarding recommendations for immunology content within undergraduate medical education curriculum. Nevertheless, every U.S. medical student is expected to achieve sufficient subject mastery to pass board exams (namely, USMLE step 1 and step 2 and COMLEX level 1 and 2 exams) and to apply the relevant knowledge to clinical care.

To support this goal, these recommendations identify the immunology concepts deemed essential for medical education and are offered as guidance, ensuring transparency and consistency while respecting each institution’s autonomy in curricular design and implementation.^20^^,^^21^ Efforts at defining core components of immunology-related curriculum have ranged from very granular definition of consensus factual concepts^22^ to broad outlines of principles and approaches.^13^ Here, we have chosen to define content LOs that medical immunology educators recommend as a foundation for medical students. Importantly, the recommended content areas align directly with the immunology domains emphasized on both the USMLE and COMLEX examinations and consistent with the highest-yield immunology material emphasized in widely used board-preparation resources. This approach allows flexibility to individual instructors regarding specific details, depth, sequence, and integration level with other elements in the curriculum while ensuring that learners gain not only the conceptual foundation required by the USMLE and COMLEX, but also the practical, testable understanding most predictive of success.

In the past, immunology was considered as a discipline primarily important in the realm of specialties such as clinical immunology/allergy, rheumatology, and infectious disease. However, with an increased understanding of the immune system’s impact on chronic disease, inflammatory conditions, and cancer biology, the importance of immunology in most clinical specialties should be appreciated. The challenge for medical immunology educators is to integrate a rapidly expanding amount of content within foundational science curricula that are generally shrinking due to the prioritization of student experiential clinical learning over classroom didactics.^23^

Based on these changing curricular models that limit the time for traditional preclerkship instruction, it was not surprising that about 25% of respondents deemed that there was insufficient time for immunology content, with as many as 17% of the polled educators reported having <20 h of available teaching time. Importantly, our survey results (Fig. 2D) highlighted a significant variation in the amount of time dedicated to immunology during the clinical phase of the curriculum (typically, the final 24–36 mo of medical school). With the proposal of vertical integration of foundational topics into the latter years of medical school curricula,^24^ it is possible that immunology topics could be integrated to a greater extent in those medical schools that have limited preclinical time. While we did not explore this aspect further in terms of curriculum organization, it deserves more attention as a potential area of expansion of formal medical immunology instruction.

The importance of curricular time is highly relevant in the context of limited premedical school exposure to immunology. Based on our survey, about half of the respondents to our survey estimate that less than 25% of their students had either taken an immunology course or engaged in immunology-related research as undergraduates. This may be an underestimate of overall exposure, due to basic immunology knowledge increasingly being taught as part of microbiology courses or other coursework (human physiology, cell biology, etc.).^7^ As immunology becomes more commonly adopted as an undergraduate course subject in biology-related and premedical majors,^6^ the hope is that some foundational topics will be solidified before entering medical school. While our data do not directly assess content complexity, the limited premedical school exposure to immunology reported by respondents suggest a challenge to medical students when they encounter immunology’s unique nomenclature and the conceptual complexities of the immune system.^25^^,^^26^

Our survey of the existing coverage of broad immunology topics/areas in medical curricula highlighted a high degree of consistency, with only transplantation and cancer immunology reportedly being covered in <90% of programs (Fig. 3A). Because of the clinical relevance and specialty-related nature of that content, this finding, in part, may reflect the coverage of the material in other courses/blocks (oncology, hematology, nephrology, pathology, etc.), rather than actual curriculum gaps. However, the importance of longitudinal preclinical curriculum supervision by trained immunologists cannot be overestimated. In contrast, we identified significantly more variation between programs in the coverage of individual subtopics (Fig. 3B), with the immunology of pregnancy, psychoneuroimmunology, and sex differences in immunity being particularly underrepresented. It is possible that these are still largely considered somewhat “exploratory” areas of immunology that have yet to demonstrate significant impact in clinical practice or treatment, but of course the same was also largely said regarding tumor immunology until a couple decades ago. As the medical impact of these topics (and potentially others) are identified, it is likely that their integration will be necessary as well to adequately prepare students for medical practice.

The LOs presented in Table 1 are thus intended to provide a set of flexible guidelines to organize and integrate modern medical immunology within the preclinical education framework and the available time. While many objectives have been left intentionally broad to promote this flexibility, future work may be necessary to better narrow specific content guidelines and prioritize knowledge areas. Similarly, the clinical conditions highlighted in Table 2 are intended to serve as a general list of conditions that highlight the importance of basic immunology information for medical practice, which is essential to engage the attention of medical students in basic science courses.^26–28^ In addition to serving as illustration of immunological concepts, immunology-related clinical content also serves the important purpose of providing material for the construction of clinically realistic scenarios for assessment, such as board-style questions, or for the design of case-based learning sessions.^29^^,^^30^

We also realize that significant constraints are imposed on individual instructors by the need to prioritize “high yield” content that is more likely to be tested on board exams, which consequently is also typically overrepresented in common external resources. This dynamic restricts curricular adaptability and slows thoughtful, forward-looking curricular revision.^31^^,^^32^ Consequently, rapidly advancing disciplines, such as immunology, are at a disadvantage as emerging concepts and evolving scientific understanding struggle to gain appropriate representation within the preclinical curriculum. To ensure that medical students are prepared both to succeed on board examinations and to practice modern medicine, it is essential that we make room for scientifically current, clinically meaningful immunology. Stronger communication and more intentional collaboration among national board organizations and the immunology education community will help align assessment with contemporary science, reduce unnecessary constraints on curricula, and ultimately support a more future-ready medical workforce. Guidelines such those presented here will provide a blueprint for more clearly aligning recommended content to existing and future high yield topics, which will be of particular importance in time constrained settings.

Our survey data (Fig. 3A) offered compelling evidence that the 15 foundational topics curated for Tables 1 and 2 represent the content most broadly endorsed by medical immunology instructors. The ordering of these topics is not prescriptive; faculty are encouraged to arrange them in a sequence that best aligns with their curriculum. Additionally, we acknowledge that certain clinical conditions and/or LOs could be presented throughout the curriculum and not simply under the foundational topics listed. For example, leukocyte adhesion deficiency was provided as an associated clinical condition under the “innate immunity and inflammation” foundational topic. However, this same clinical condition could have been listed under the “immunodeficiency” foundational topic if the instructor is teaching a block of immunodeficiencies at the same time within a curriculum. The goal of Tables 1 and 2 was not only to provide LOs and clinical conditions, but also to imply the flexibility with which each topic could be presented.

Among the limitations to this study are possible biases due to the response rate. However, the number, diversity of geographic distribution of participating institutions, and breakdown of MD versus DO schools would be expected to provide adequate representative feedback across a broad range of immunology instructors. Another possible criticism is that these recommendations could limit the range of immunology topics presented to medical students. Although the effort was made to be as comprehensive as possible in our recommendation and to include as many respondent suggestions as possible, we recognize that a large part of curriculum design consists of prioritizing content, and our recommendation are not intended to be prescriptive.

Analysis of the free-response entries from both surveys revealed a shared theme, illustrated by the following quote, “This is an important effort that provides a standardized framework ensuring consistency and relevance in medical education. By integrating fundamental immunological advances and clinical relevance, these guidelines help align curriculum with the continuously evolving discoveries of the immune system role in various diseases. The ongoing revisions by the AAI society-backed task force will promote continuous improvement and equity across the U.S. institutions, ultimately strengthening the preparation of future physician scientists.”

These recommendations provide guidance to both experienced and new instructors of medical immunology, contribute to a framework for curriculum development, and ultimately contribute to a focused attention on immunology learning to adequately prepare medical students for the future of immunology within the practice of medicine.

## Conclusions

The collaborative effort of the AAI Medical Education Immunology Curriculum Task Force resulted in a dynamic resource designed for ongoing refinement. The AAI Education Committee will revisit and update this document regularly to ensure that its content remains current, relevant, and reflective of advances in the field. This initiative demonstrates how a committed team of educators, united by a shared vision, came together to create an innovative tool that strengthens immunology education design and enhances instructional practices. Ultimately, the Task Force aims for these immunology content recommendations to serve as a clear, expert-driven guide that empowers educators to build rigorous, forward-looking curricula for the next generation of medical trainees.

## Supplementary Material

vlag041_Supplementary_Data

## Data Availability

The data underlying this article are available in the article and in its online supplementary material.
