# Supplementary material for: Status of immunology education and establishment of American Association of Immunologists–recommended immunology content guidelines for undergraduate medical education in the United States
Source: Immunohorizons. 2026 Aug 20;10(8):vlag041. doi: 10.1093/immhor/vlag041 (PMC13493048; doi:10.1093/immhor/vlag041)

Supplementary Files

Supplemental Figure 1. Survey 1
The survey evaluated foundational topics and subtopics covered, hours dedicated to instruction, and perceived adequacy of immunology preparation for clinical training within undergraduate medical school immunology education.

University of Colorado **Denver**

Please share your name and institution

Which type of degree does your institution offer?

- ☐ MD
- ☐ DO

How is the immunology content delivered in your curriculum? Select all that apply.

- ☐ Standalone course/block
- ☐ Combined course/block (e.g., microbiology and immunology)
- ☐ Content is integrated throughout the curriculum
- ☐ Other (please describe)

When is the immunology content delivered? Please select the most appropriate answer.

- ○ Content within the first year only
- ○ Content within the second year only
- ○ Content primarily in pre-clerkship years
- ○ Content within the first year and beyond
- ○ Across all years
- ○ Other (please describe)

How much instructional time is devoted to immunology content in the **preclinical** year(s)?

Please provide an answer in units of contact hours selecting the most appropriate checkbox below. Best estimates are okay. Please select the most appropriate answer.

- ○ 0-10 hours
- ○ 10-20 hours
- ○ 20-30 hours
- ○ 30-40 hours
- ○ 40-50 hours
- ○ 50+ hours

Additional contextual information such as the number of days, weeks, etc. over which immunology is taught in the preclinical year(s) would also be incredibly beneficial and can be added in the free response below.

How much instructional time is devoted to immunology content in the **clinical** years?

Please provide an answer in units of contact hours selecting the most appropriate checkbox below. Best estimates are okay. Please select the most appropriate answer.

- ○ 0-10 hours
- ○ 10-20 hours
- ○ 20-30 hours
- ○ 30-40 hours
- ○ 40-50 hours
- ○ 50+ hours

Additional contextual information such as the number of days, weeks, etc. over which immunology is taught in the clinical years would also be incredibly beneficial and can be added in the free response below.

Who covers foundational immunology teaching at your institution. Does your institution have at least 1 trained immunologist teaching foundational immunology content?

- ○ Trained Immunologist
- ○ Covered by non-Immunologist
- ○ Mixture of trained Immunologist and non-Immunologist

Using the following scale, where **0** represents not at all sufficient and **10** represents more than sufficient, do you (and your team) feel that you can cover essential immunology content in the time that you are provided?

0	2	4	6	8	10
○	○	○	○	○	○

Do you have a feeling for the proportion of your students who have substantive prior exposure to immunology (e.g., an undergraduate course), research experience, prior degree in the field, etc.?

Please select the most appropriate percentage range listed below. Best estimates are okay.

- ○ >75%
- ○ 50-74%
- ○ 25-49%
- ○ <25%
- ○ I have no idea

When was the last time that your institution substantially revised or renovated the medical school curriculum? Please select the most appropriate answer. Best estimates are okay.

- ○ 1-2 years ago
- ○ 3-5 years ago
- ○ 6-8 years ago
- ○ 9+ years ago

Of the 12 foundational immunology topics listed below, please select 'yes' or 'no' if they are included or not in the curriculum during the undergraduate medical education preclinical year(s)?

	Yes	No
Cells, Organs and Microenvironments of the Immune System	○	○
Innate Immunity and Inflammation	○	○
Complement	○	○
Host-Pathogen Interaction	○	○
Adaptive Immunity - MHC and T cells	○	○
Adaptive Immunity - B cells and Antibody	○	○
Vaccines - Formulations and Immune Response	○	○
Type I-IV Hypersensitivity	○	○
Transplantation Immunology	○	○
Cancer Immunology	○	○
Tolerance and Autoimmunity	○	○
Immunodeficiency	○	○

Within the systems listed below, please select if the larger system is included or not in the curriculum during the undergraduate medical education preclinical year(s).

Further, under each system, please select if the individual topics are included or not in the curriculum as well.

- ☐ Mucosal Immunology
- ☐ Immunology and the GI Tract
- ☐ Immunology and the Endocrine System
- ☐ Immunology and MSK
- ☐ Immunology and the Nervous System
- ☐ Immunology and the Renal/Urinary System
- ☐ Immunology and the Pulmonary System
- ☐ Immunology and the Cardiovascular System
- ☐ Immunology and the Skin
- ☐ Immunology and the Reproductive System
- ☐ Immunology and Pregnancy
- ☐ Blood Group Reactions
- ☐ Sex Differences in Immune Responses
- ☐ Diagnostic Laboratory Immunology / Basic Analytical Techniques
- ☐ Immune System Across the Lifespan
- ☐ Psychoneuroimmunology

NOTE. If they check the boxes above, they will get additional questions below – see next pages

Within the systems listed below, please select if the larger system is included or not in the curriculum during the undergraduate medical education preclinical year(s).

Further, under each system, please select if the individual topics are included or not in the curriculum as well.

- [x] Mucosal Immunology
- [x] Immunology and the GI Tract
- [x] Immunology and the Endocrine System
- [x] Immunology and MSK
- [x] Immunology and the Nervous System
- [x] Immunology and the Renal/Urinary System
- [x] Immunology and the Pulmonary System
- [x] Immunology and the Cardiovascular System
- [x] Immunology and the Skin
- [x] Immunology and the Reproductive System
- [x] Immunology and Pregnancy
- [x] Blood Group Reactions
- [x] Sex Differences in Immune Responses
- [x] Diagnostic Laboratory Immunology / Basic Analytical Techniques
- [x] Immune System Across the Lifespan
- [x] Psychoneuroimmunology

Mucosal Immunology: Sub Topics

- ☐ Respiratory tract
- ☐ Gastrointestinal tract
- ☐ Urogenital tracts
- ☐ Other

Immunology and GI Tract: Sub Topics

- ☐ Inflammatory Bowel Disease (IBD)
- ☐ Celiac Disease
- ☐ Food Allergies
- ☐ Other

Immunology and the Endocrine System: Sub Topics

- ☐ Type 1 Diabetes
- ☐ Hashimoto's Disease
- ☐ Graves Disease
- ☐ Other

Immunology and MSK: Sub Topics

- ☐ Arthritis
- ☐ Myositis
- ☐ Other

Immunology and the Nervous System: Sub Topics

- ☐ Multiple Sclerosis (MS)
- ☐ Guillain-Barre Syndrome (GBS)
- ☐ Myasthenia gravis
- ☐ Other

Immunology and the Renal/Urinary System: Sub Topics

- ☐ Systemic Lupus Erythematosus (SLE)
- ☐ Anti-Glomerular Basement Membrane (GBM) Disease
- ☐ Other

Immunology and the Pulmonary System: Sub Topics

- ☐ Asthma
- ☐ Anaphylaxis
- ☐ Autoimmune Lung Disease
- ☐ Other

Immunology and the Cardiovascular System: Sub Topics

- ☐ Atherosclerosis
- ☐ Vasculitis
- ☐ Other

Immunology and the Skin: Sub Topics

- ☐ Eczema (Atopic dermatitis)
- ☐ Psoriasis
- ☐ Vitiligo
- ☐ Pemphigus vulgaris
- ☐ Other

Immunology and the Reproductive System: Sub Topics

- ☐ Blood-Gonad-Barriers
- ☐ uNK cells
- ☐ Other

Immunology and Pregnancy: Sub Topics

- ☐ Maternal-Fetal Interface
- ☐ Rh incompatibility
- ☐ Other

Blood Group Reactions: Sub Topics

- ☐ Transfusion reactions
- ☐ Fetal/Newborn Hemolytic Disease
- ☐ Other

Sex Differences in Immune Responses: Sub Topics

- [ ] Other

Diagnostic Laboratory Immunology / Basic Analytical Techniques: Sub Topics

- [ ] ELISA
- [ ] ELISPOT
- [ ] Flow Cytometry
- [ ] Other

Immune System Across the Lifespan: Sub Topics

- [ ] Development through Immunosenescence
- [ ] Other

Psychoneuroimmunology: Sub Topics

- [ ] Other

Please use this free response area to list topics that were missing or other useful details related to immunology topics and curriculum you desire to provide.

University of Colorado **Denver**

THANK YOU for participating in this survey.
Your time and expertise are valuable to this project.

Supplemental Figure 2. Survey 2
The survey further evaluated foundational topics and subtopics covered, associated clinical conditions, and perceived adequacy of immunology preparation for clinical training within undergraduate medical school immunology education.

University of Colorado **Denver**

Thank you for providing your review feedback on the AAI Medical Immunology Content Recommendations. Please note that the following survey can be completed in multiple sittings as your progress is saved.

→

University of Colorado **Denver**

Review Table 1: Foundational topics and associated learning objectives for immunology medical education (attached/linked here).

Rate on a scale of 1-5, with 1 being little coverage and 5 being full coverage, how representative you feel the foundational topics are in terms of coverage at your institution.

1 2 3 4 5

Rate on a scale of 1-5, with 1 being insufficient and 5 being fully sufficient, how comprehensive the Table 1 learning objectives are relative to a medical education environment. (keeping in mind the breadth of specialty outcomes for medical students!)

1 2 3 4 5

Please provide narrative feedback on particular improvements, again keeping in mind the diversity of medical student career trajectories. For example, are there learning objectives that you would add? Are there learning objectives that you would eliminate from the existing list in Table 1?

University of Colorado **Denver**

Review Table 2: Foundational Topics and Associated Subtopics for Immunology Medical Education (attached/linked here).

Please keep in mind that Table 1 is the base and Table 2 is meant to add more granular possibilities depending on each user's unique curricular circumstances. Our intent with Table 2 is to provide a more inclusive menu of possibilities for one to select in developing/refining medical immunology content.

Are there any specific subtopics/sub-LOs that you believe should be edited? Please provide your recommendations here. A bullet list is appreciated.

→

University of Colorado **Denver**

Review Table 3: Foundational Topics and Associated Conditions for Immunology Medical Education. (attached/linked here)

Please keep in mind that Table 1 is the base, and Table 3 is meant to provide a list of conditions aligned to Foundational Topics that an immunology educator might highlight. Our intent with Table 3 is to provide a more inclusive list; the Task Force would especially appreciate further community additions.

Are there any specific conditions that you believe should be edited (e.g., language, re-alignment, or additions)? Please provide your recommendations here. A bullet list is appreciated.

→

University of Colorado **Denver**

The hard part is over! There are just a few more quick questions remaining to wrap up this survey.

Rate on a scale of 1-5, with 1 being not helpful at all and 5 being very helpful, how you feel that this society-endorsed content recommendation will be in the development and continuing revision of immunology teaching in medical schools.

1 2 3 4 5

Please share any additional thoughts about this effort below.

If you would like to be acknowledged in the eventual paper about these recommendations, please type in your full name, credentials and institution as you would like them to appear.

→

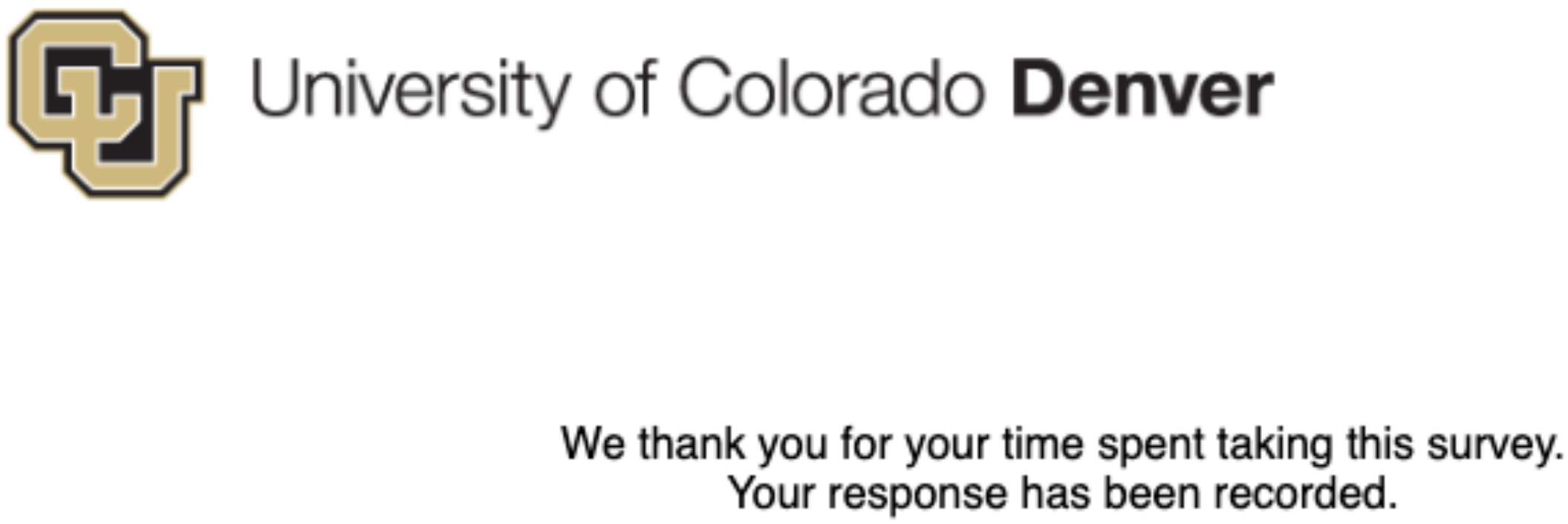

Supplemental Figure 3. Geographic Distribution of Survey 1 and 2 Respondents
Map of the United States depicting respondent locations for two independent surveys. Green markers indicate respondents to Survey 1, and blue markers indicate respondents to Survey 2. Both surveys demonstrate broad geographic representation, with respondents throughout the U.S.A., including the West Coast, Midwest, South, and Northeast.

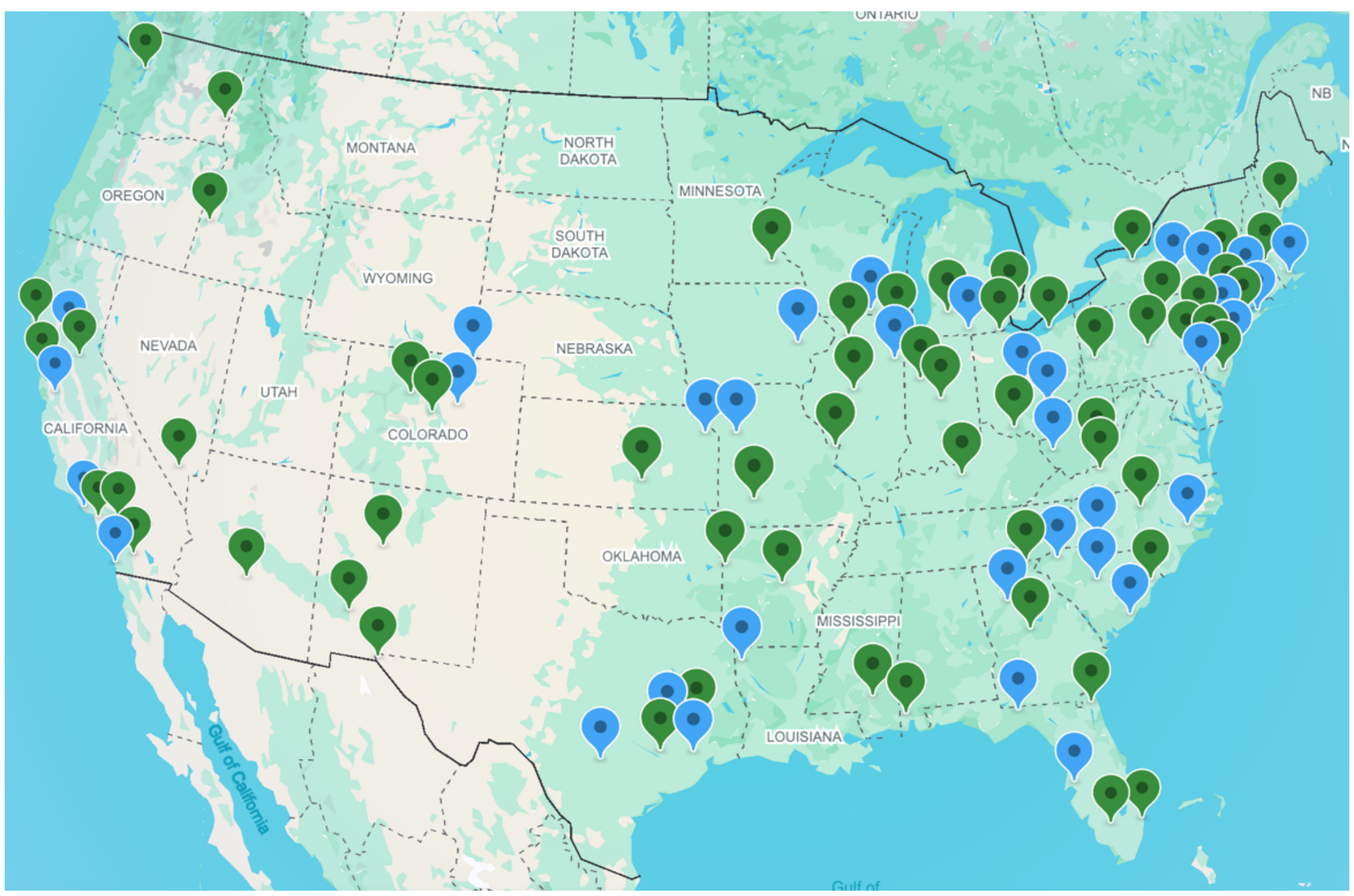

Supplemental Figure 4. Evaluation of Proposed Foundational Topics and Associated Learning Objectives
Responses as percentage of total for the following survey questions: A) Rate of a scale of 1 (little coverage) to 5 (full coverage) how representative the table 1 foundational topics are in terms of

coverage at your institution. B) Rate on a scale of 1 (insufficient) to 5 (fully sufficient) how comprehensive the table 1 learning objectives are relative to a medical education environment.

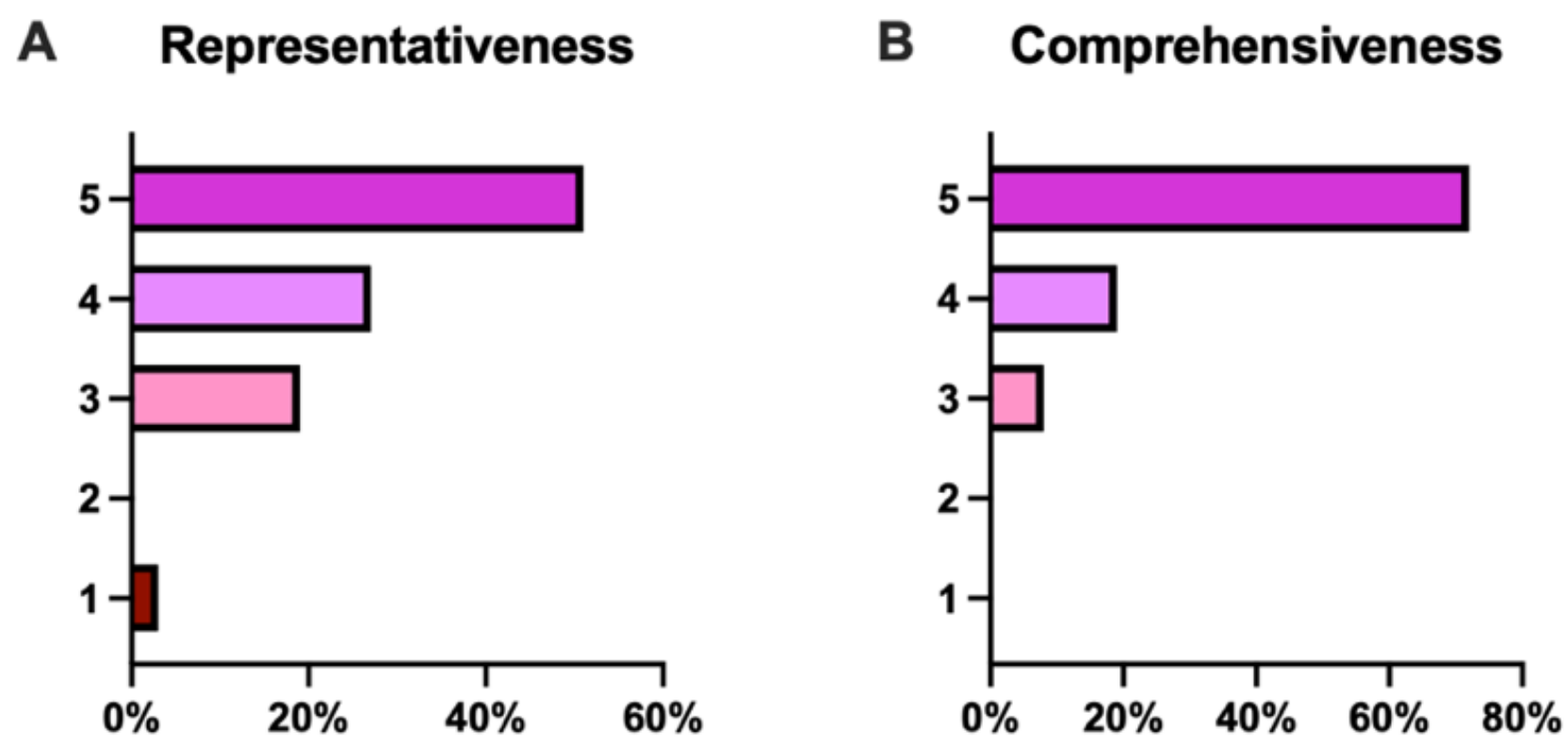

Supplement: Supplementary file 1 [file vlag041_supplementary_data.pdf]
